# Supplementary material for: Structure of the central Staphylococcus aureus AAA+ protease MecA/ClpC/ClpP
Source: Commun Biol. 2025 Oct 14;8:1467. doi: 10.1038/s42003-025-08908-w (PMC12521514; doi:10.1038/s42003-025-08908-w)

Supplementary material

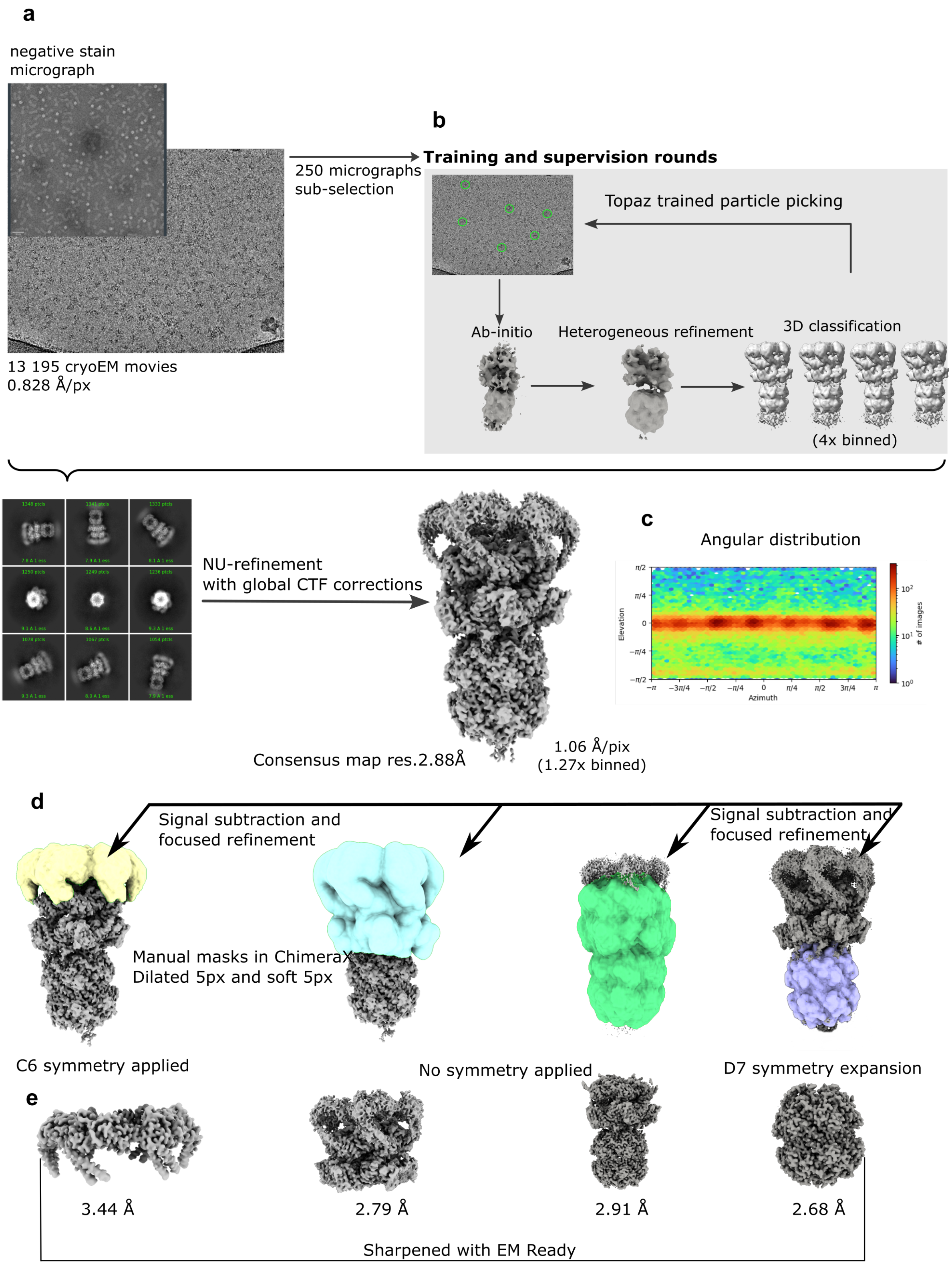

Supplementary Figure 1: Cryo-EM processing workflow of the S. aureus MecA/ClpC/ClpP dataset (a) Example micrographs of negatively stained and vitrified

complex. **(b)** Particle picking was performed with Topaz[76] with iterations of training, supervision and abinition 3D determination. **(c)** 2D classification and 3D refinement rounds were performed until a subset of particles with good angular distribution was selected. A consensus map at 2.9Å resolution was obtained **(d)** 3D masking and classification was performed to resolve the best possible map for each part of the complex. The maps were postprocessed using EMReady[51]. **(e)** Locally refined and sharpened bodies of MecA crown, MecA/ClpC, ClpC/ClpP and ClpP with appropriate symmetry applied.

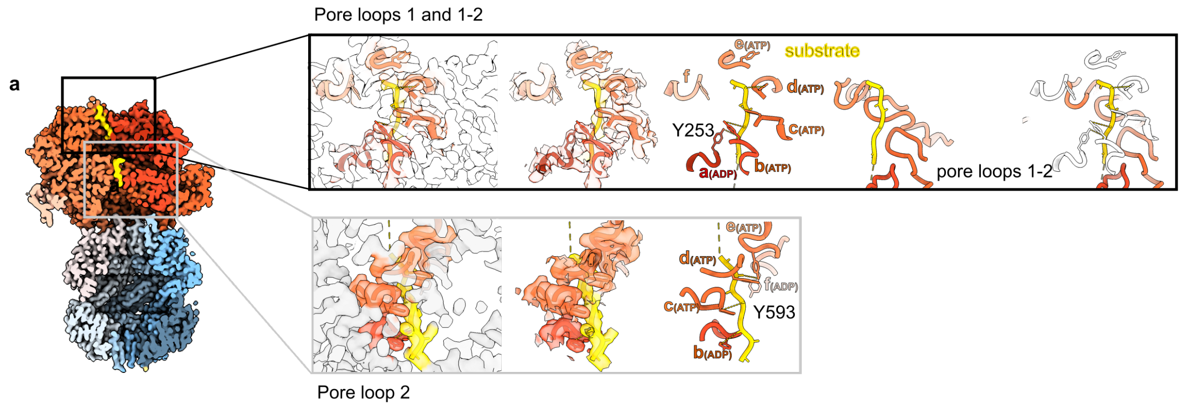

Supplementary Figure 2: Substrate binding to ClpC pore loops in the MecA/ClpP/ClpC complex. (a) Cryo-EM density is shown. Pore loops were modelled only where density is clearly visible. In both AAA+ rings pore loops are arranged in a staircase. Two 8 aa-long substrate stretches are visible in both rings. For AAA-1 primary pore-loop 1 and secondary pore-loop 1-2 are shown.

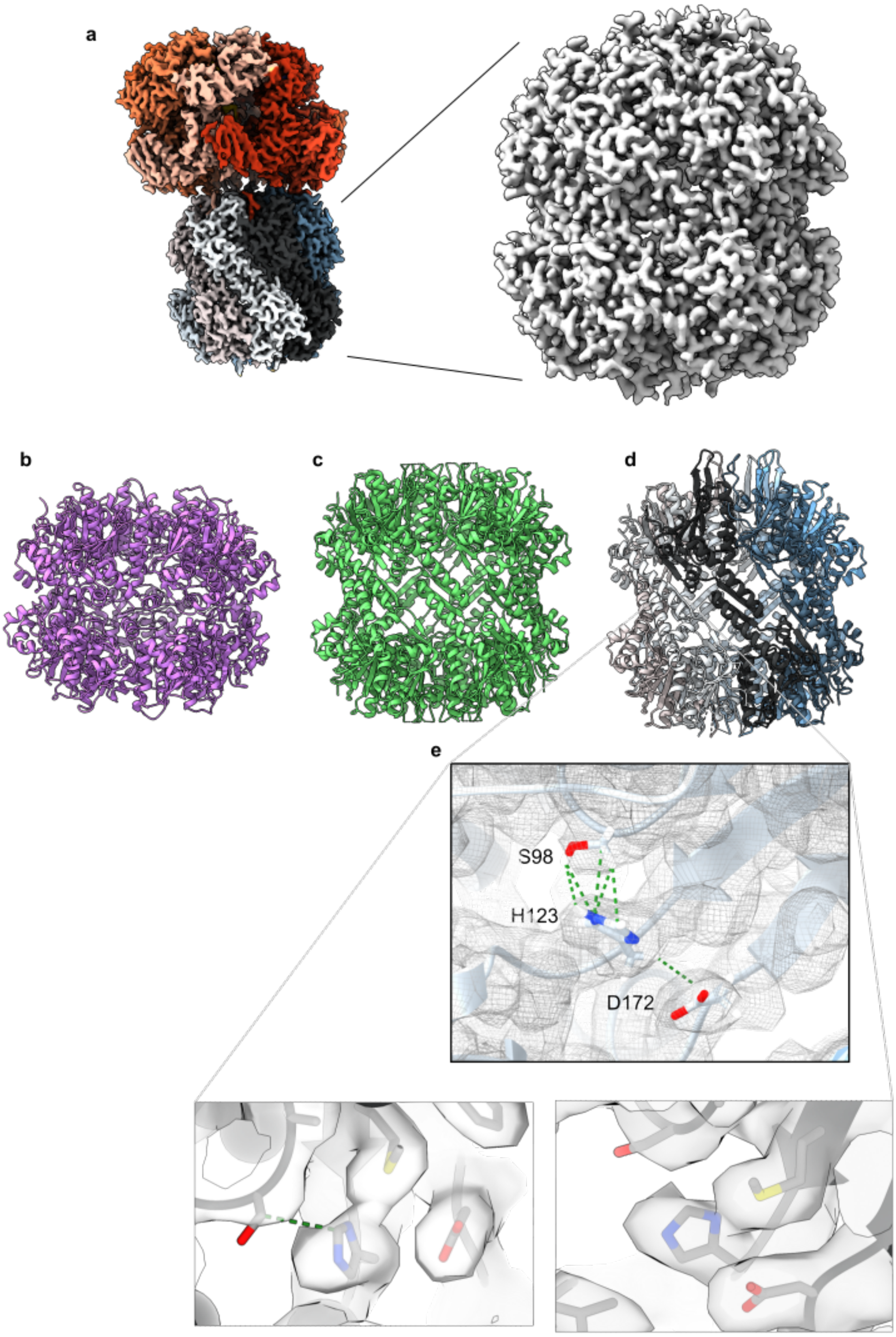

Supplementary Figure 3: The *S. aureus* ClpP peptidase (a) Cryo-EM map of the ClpP tetradecameric body of the MecA/ClpC/ClpP complex, with D7 applied symmetry. **(b)** Compact inactive conformation of *S. aureus* ClpP as in the crystal structure with PDB code 3st9[82]. **(c)** Extended active conformation of S. aureus ClpP as in the crystal structure with PDB code 3v5e[83] and **(d)** as in the current study in complex with MecA and ClpC. **(d)** Position of the catalytic triad for proteolysis in the ClpP/ClpC complex with shown surfaces.

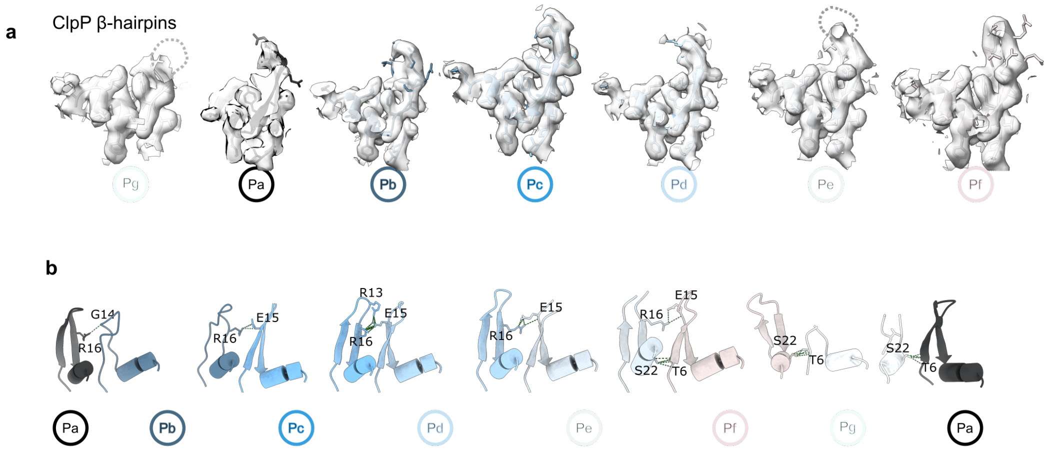

Supplementary Figure 4: The *S. aureus* ClpP NTD β-hairpins (a) Region encompassing the β-hairpins (residues 1-20) of each ClpP protomer, the density is shown and it is visible for protomers **Pa, Pb, Pc, Pd** and **Pf,** although at different level of side-chain resolution. Density for the loops of protomers **Pg** and **Pe** is missing. **(b)** Lines represent hydrogen and salt bridge contacts between residues of adjacent ClpP protomers β-hairpins.

Supplementary figure 5a

```
Saureus_ClpC
                        1        10         20         30         40         50
Saureus_ClpC            MLFGRLTERAQRVLAHAQEEAIRLNHSNIGTEHLLLGLMKEP...EGIAAKVLESFNITE
Bsubtilis_ClpC          MMFGRFTERAQKVLALAQEEALRLGHNNIGTEHILLGLVREG...EGIAAKALQALGLGS
Mtubercolosis_ClpC      .MFERFTDRARRVVVLAQEEARMLNHNYIGTEHILLGLIHEG...EGVAAKSLESLGISL
Ecoli_ClpA              ....MLNQELELSLNMAFARAREHRHEFMTVEHLLLALLSNPSAREALEACSVDLVALRQ
Senterica_ClpA          ....MLNQELELSLNMAFARAREHRHEFMTVEHLLLALLSNPSAREALEACSVDLVALRQ
Spneumoniae_ClpE        ...............MLCQNCKINDSTIHLYTNLNGKQKQI...DLCQNCYKIIKTDPN
Efaecium_ClpE           ...............MLCQNCGKNEATIHLYATVNGQRTQL...DYCQSCYQKIKNQQN
Ecoli_ClpX              ............................................................
Mtuberculosis_CLpX      ............................................................

Saureus_ClpC
                          60        70         80         90        100        110
Saureus_ClpC            DKVIEEVEKLIGHGQ.DHVGTLHYTPRAKKVIELSMDEARKLHHNFVGTEHILLGLIREN
Bsubtilis_ClpC          EKIQKEVESLIGRGQ.EMSQTIHYTPRAKKVIELSMDEARKLGHSYVGTEHILLGLIREG
Mtubercolosis_ClpC      EGVRSQVEEIIGQGQQAPSGHIPFTPRAKKVLELSLREALQLGHNYIGTEHILLGLIREG
Ecoli_ClpA              ELEAFIEQTTPVLPASEEERDTQPTLSFQRVLQRAVFHVQSSGRNEVTGANVLVAIFSEQ
Senterica_ClpA          ELEAFIEQTTPVLPASEEERDTQPTLSFQRVLQRAVFHVQSSGRSEVTGANVLVAIFSEQ
Spneumoniae_ClpE        NSLFKGMTDLNNRDFDPFGDFFNDLNNFRPSSNTPPIPPTQSGGGYGGNGGYGS......
Efaecium_ClpE           GGLTNMAQNDPFGFGSLDDLFRSMSRQMQQQGNYEQTPPTQFGGNNNFNGGQ........
Ecoli_ClpX              ............................................................
Mtuberculosis_CLpX      ............................................................

                                                                          α1    β1
Saureus_ClpC
                          120       130        140                 150       160
Saureus_ClpC            EGVAARVFANLDLNITKARAQVVKALG........NPEMSNKNAQASKSNNTPTLDSLAR
Bsubtilis_ClpC          EGVAARVLNNLGVSLNKARQQVLQLLG........SNETGSSAAGTNSNANTPTLDSLAR
Mtubercolosis_ClpC      EGVAAQVLVKLGAELTRVRQQVIQLLSGYQGKEAAEAGTGGRGGESGSPSTSLVLDQFGR
Ecoli_ClpA              ESQAAYLLRKHEVSRLDVVNFISHGTR..KDEPTQSSDPGSQPNSEEQAGGEERMENFTT
Senterica_ClpA          ESQAAYLLRKHEVSRLDIVNFISHGTR..KDEPSQSSDLGNQPTGDEQAGGEERMENFTT
Spneumoniae_ClpE        ....................................QNRGSAQTPPPSQEKGLLEEFGI
Efaecium_ClpE           .............................................PPQGNTEGLLGEYGI
Ecoli_ClpX              ............................................................
Mtuberculosis_CLpX      ............................................................

                            α2              α3               β2         α4
Saureus_ClpC
                        170        180        190        200        210        220
Saureus_ClpC            DLTVIAKDGTLDPVIGRDKEITRVIEVLSRRTKNNPVLIGEPGVGKTAIAEGLAQAIVNN
Bsubtilis_ClpC          DLTAIAKEDSLDPVIGRSKEIQRVIEVLSRRTKNNPVLIGEPGVGKTAIAEGLAQQIINN
Mtubercolosis_ClpC      NLTAAAMEGKLDPVIGREKEIERVMQVLSRRTKNNPVLIGEPGVGKTAVVEGLAQAIVHG
Ecoli_ClpA              NLNQLARVGGIDPLIGREKELERAIQVLCRRRKNNPLLVGESGVGKTAIAEGLAWRIVQG
Senterica_ClpA          NLNQLARVGGIDPLIGREKELERAIQVLCRRRKNNPLLVGESGVGKTAIAEGLAWRIVQG
Spneumoniae_ClpE        NVTEIARRGDIDPVIGRDDEIIRVIEILNRRTKNNPVLIGEPGVGKTAVVEGLAQKIVDG
Efaecium_ClpE           NITEQARQGDIDPVIGRDDEIKRVIEILNRRTKNNPVLIGEPGVGKTAVVEGLAQKIVDG
Ecoli_ClpX              .............MTDKRKDGSGKLLYCSFCGKSQHEVRKLIAGPSVYICDECVDLCND
Mtuberculosis_CLpX      ............................................................
                                                              WalkerA

                                η1     β3     α5             α6              β4    η2
Saureus_ClpC
                        230        240        250        260        270        280
Saureus_ClpC            EVPETLKDKRVMSLDMGTVVAGTKYRGEFEERLKKVMEEIQQAGNVILFIDELHTLVGAG
Bsubtilis_ClpC          EVPEILRDKRVMTLDMGTVVAGTKYRGEFEDRLKKVMDEIRQAGNIILFIDELHTLIGAG
Mtubercolosis_ClpC      EVPETLKDKQLYTLDLGSLVAGSRYRGDFEERLKKVLKEINTRGDIILFIDELHTLVGAG
Ecoli_ClpA              DVPEVMADCTIYSLDIGSLLAGTKYRGDFEKRFKALLKQLEQDTNSILFIDEIHTIIGAG
Senterica_ClpA          DVPEVMADCTIYSLDIGSLLAGTKYRGDFEKRFKALLKQLEQDTNSILFIDEIHTIIGAG
Spneumoniae_ClpE        DVPHKLQGKQVIRLDVVSLVQGTGIRGQFEERMQKLMEEIRKREDIILFIDEIHEIVGAG
Efaecium_ClpE           DVPQKLMDKEVIRLDVVSLVQGTGIRGQFEERMQKLIEEIRQAENVILFIDEVHEIVGAG
Ecoli_ClpX              IIREEIK.......EVAPHRERSALPTPHEIRNHLDDYVIGQEQ................
Mtuberculosis_CLpX      ..................ELPKPAEIREFLEGYVIGQDT.....................
                                         pore-loop 1               Walker B     pore loop 1-2

                                       α7          β5     α8         α9     β6
Saureus_ClpC             TT
                        290        300        310        320        330  R-finger 340
Saureus_ClpC            .GAEGAIDASNILKPALARGELQCIGATTLDEYRKNIEKDAALERRFQPVQVDEPSVVDT
Bsubtilis_ClpC          .GAEGAIDASNILKPSLARGELQCIGATTLDEYRKYIEKDAALERRFQPIQVDQPSVDES
Mtubercolosis_ClpC      .AAEGAIDAASILKPKLARGELQTIGATTLDEYRKYIEKDAALERRFQPVQVGEPTVEHT
Ecoli_ClpA              AASGGQVDAANLIKPLLSSGKIRVIGSTTYQEFSNIFEKDRALARRFQKIDITEPSIEET
Senterica_ClpA          AASGGQVDAANLIKPLLSSGKIRVIGSTTYQEFSNIFEKDRALARRFQKIDITEPSVEET
Spneumoniae_ClpE        SASDGNMDAGNILKPALARGELQLVGATTLNEYR.IIEKDAALERRMQPVKVDEPTVDET
Efaecium_ClpE           SAGDGNMDAGNILKPALARGELQMVGATTLNEYR.IIEKDAALERRMQPVRVDEPTVEET
Ecoli_ClpX              ................AKKVLAVAVYNHYKRLRNGDTSNG.....VELGKSNILLI
Mtuberculosis_CLpX      ................AKRTLAVAVYNHYKRIQAGEKGRDSRCEPVELTKSNILML
```

Supplementary figure 5a

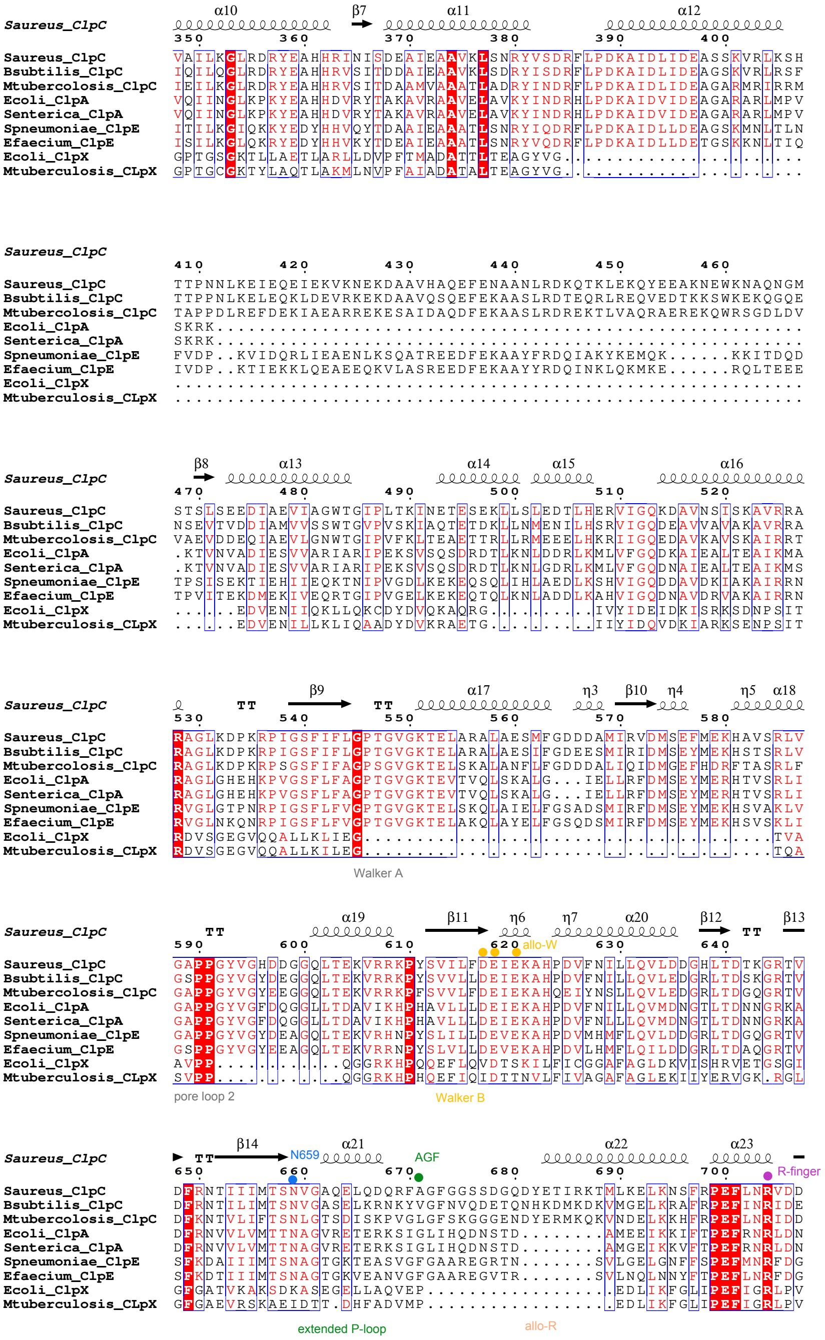

Supplementary figure 5a

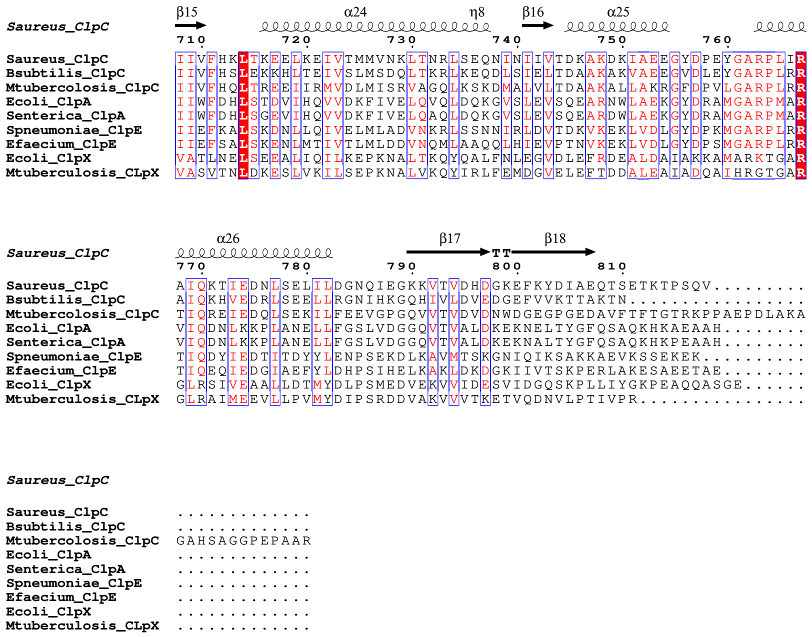

Supplementary figure 5b

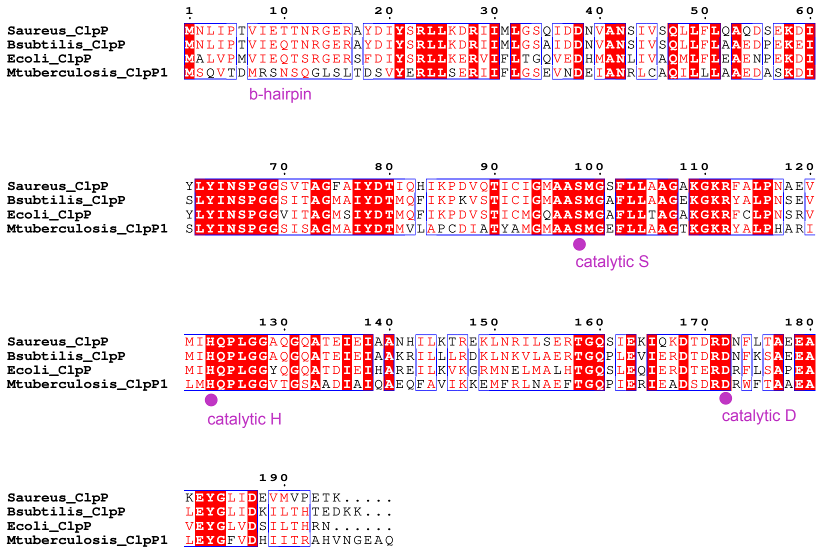

Supplementary Figure 5: Sequence alignment of **(a)** ClpC with ClpA, ClpE and ClpX from different Bacteria. Positions of Walker A and B motifs and R-finger motifs, pore-loops and allo-W, alloR and extended P-loops are indicated. **(b)** ClpP with the β-hairpin and the catalytic triad (S/H/D) indicated

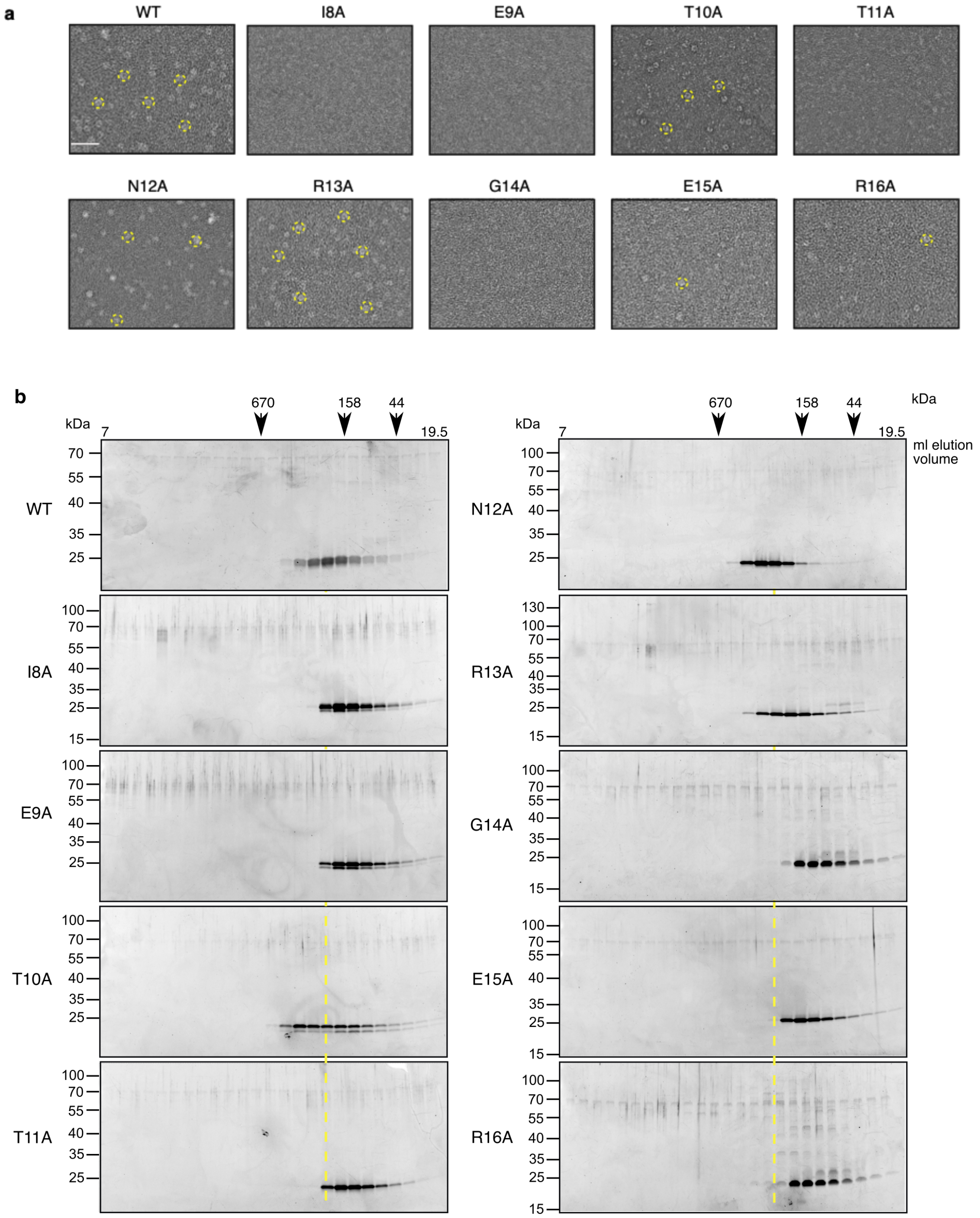

Supplementary Figure 6: ClpP oligomerization figure ClpP β-hairpin mutants exhibit diverse oligomerization defects. (**a**) Negative-stain micrographs of ClpP wild type (WT) and indicated β-hairpin mutants. Selected barrel-like particles are indicated. Scale bar, 50 nm. (**b**) Oligomeric state of ClpP WT and mutants were determined by size-exclusion

chromatography. Elution fractions were analyzed by SDS-PAGE. The peak elution positions of ClpP WT (dashed line) and of a protein standard (arrows) are indicated.

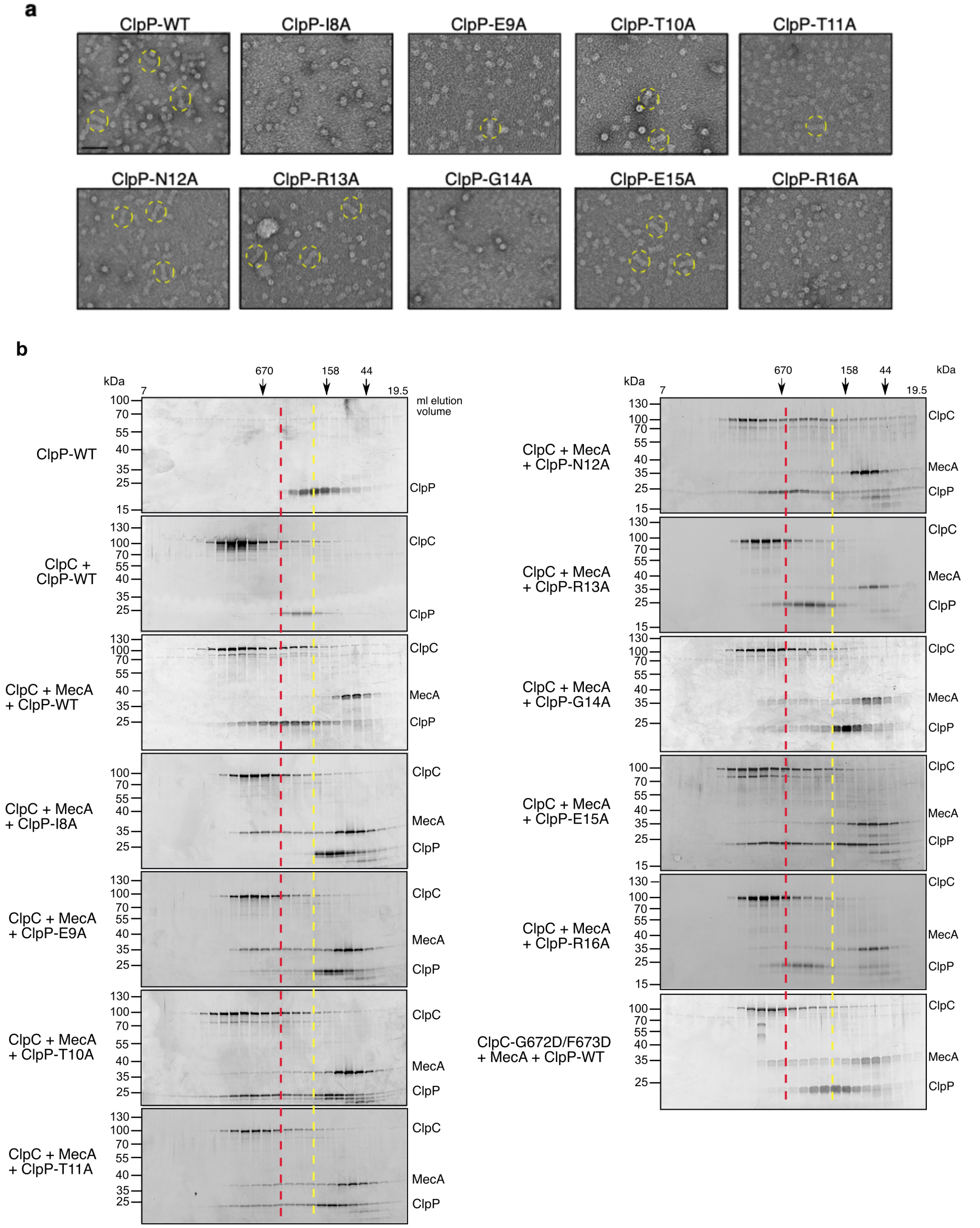

Supplementary Figure 7: ClpC/ClpP complex formation ClpP β-hairpin mutants are differently affected in complex formation with ClpC. Complex formation between ClpP (WT or β-hairpin mutants) and ClpC (WT or P-loop mutant G672D/F673D) was analyzed in absence or presence of MecA by size-exclusion chromatography as indicated. Elution fractions were analyzed by SDS-PAGE. Peak elution positions of ClpP WT in absence or presence of MecA/ClpC are indicated by yellow and red dashed lines. Peak elution positions of a protein standard are indicated by arrows.

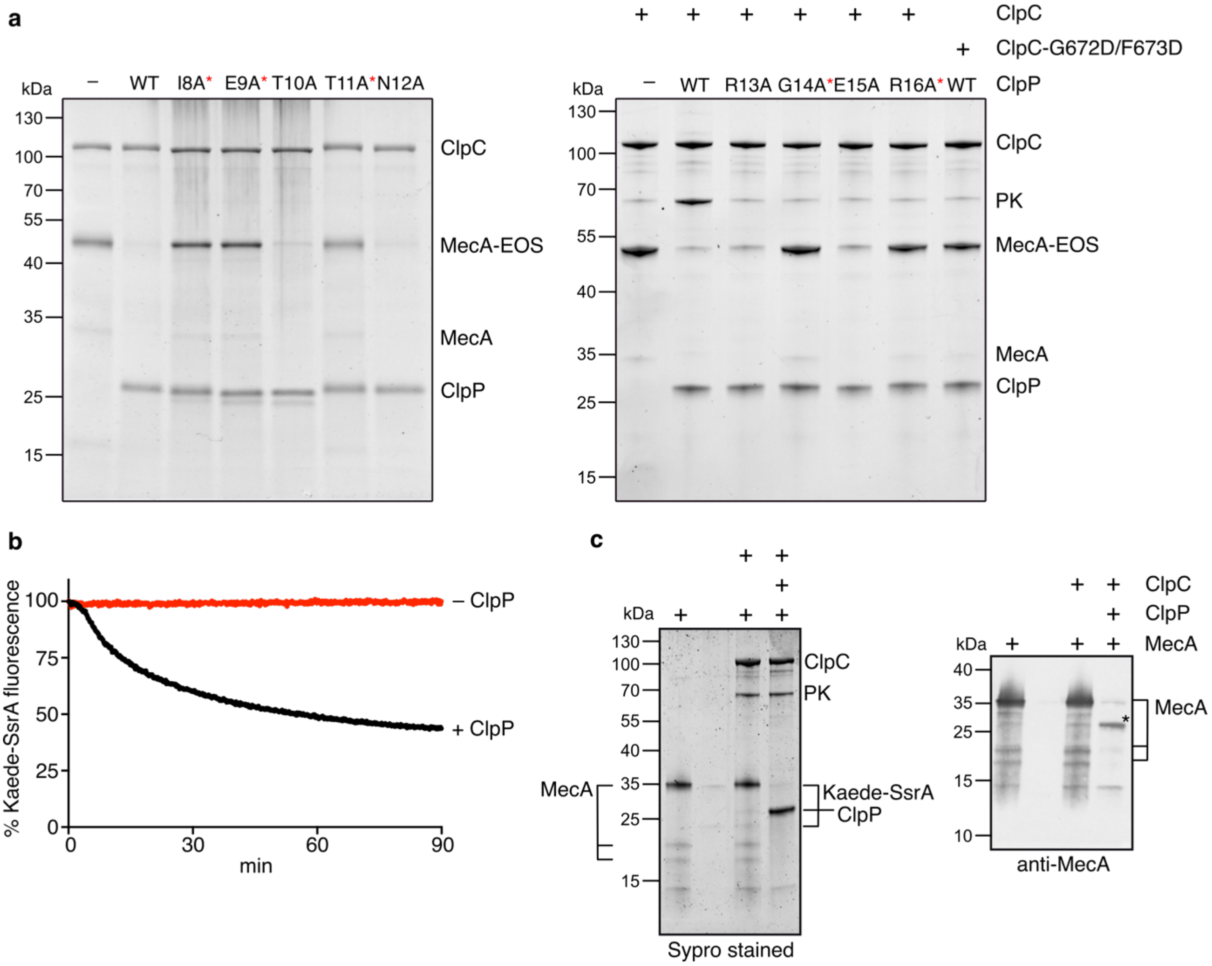

Supplementary Figure 8: ClpP stimulation of ClpC unfolding activity. (a) MecA-mEOS3.2 was incubated in presence of ClpC (WT or P-loop mutant G672D/F673D) and ClpP (WT or β-hairpin mutants) for 30 min in presence of an ATP regenerating system. Degradation reactions were analyzed by SDS-PAGE and positions of proteins are indicated (PK: Pyruvate kinase, MecA: co-purifying cleavage product of His_6-MecA-mEOS3.2). ClpP β-hairpin mutants that are defective in complex formation are labelled with *. **(b)** Unfolding of Kaede-SsrA by MecA/ClpC was monitored in absence or presence of ClpP. Initial Kaede-SsrA fluorescence was set as 100%. Samples of the same reactions (90 min) were analyzed **(c)** by SDS-PAGE and western blot analysis using MecA-specific antibodies. MecA and Kaede-SsrA samples are provided as reference. Protein positions are indicated. *: antibody crossreation with ClpP.

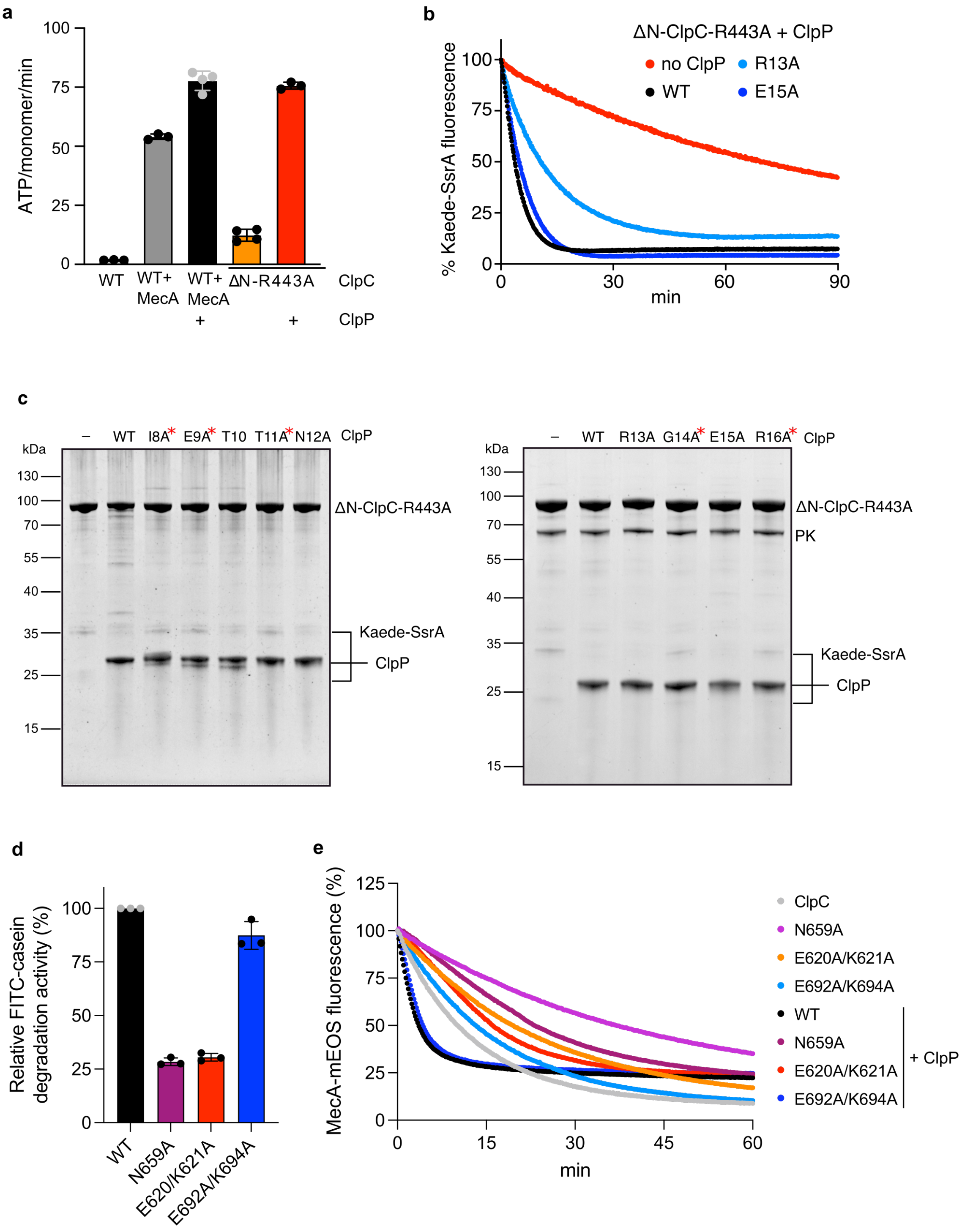

Supplementary Figure 9: ClpP enhances of ATPase and threading activity of a MecA-independent ClpC mutant. (a) ATPase activities of ClpC WT or ΔN-ClpC-R443A were determined in the presence of MecA and ClpP as indicated. **(b)** Unfolding of Kaede-SsrA by ΔN-ClpC-R443A was monitored in the absence or presence of ClpP WT and β-hairpin mutants. Initial Kaede-SsrA fluorescence was set as 100%. **(c)** Kaede-SsrA was incubated in presence of ΔN-ClpC-R443A and ClpP (WT or β-hairpin mutants) for 90 min in presence of an ATP regenerating system. ClpP β-hairpin mutants that are defective in complex formation are labelled with *. Degradation reactions were analyzed by SDS-PAGE and positions of proteins are indicated (PK: Pyruvate kinase). **(d)** FITC-casein

degradation rates (% fluorescence increase/min) were determined for ClpC WT and indicated mutants in presence of ClpP WT. The proteolytic activity of ClpC-WT was set as 100%. (**e**) Unfolding of MecA-mEOS3.2 by ClpC WT or mutants was monitored in the absence or presence of ClpP WT. Initial MecA-mEOS3.2 fluorescence was set as 100%. Error bars represent standard deviations (n=3)(a,d).

Supplementary video 1: Motions of the MecA/ClpC/ClpP complex's bodies (MecA crown, ClpC/ClpP and ClpP protease bodies) relative to each other. Animation of the volumes obtained via 3D variability analysis. Suggested to play at 0.5x.

Supplementary Table 1: ***Characterization of S. aureus ClpP β-hairpins mutants.***

ClpP variant	ClpP 14-mer formation		MecA/ClpC/ClpP complex formation		ClpP proteolytic activity			MecA/ClpC/ClpP proteolytic activity		ClpC ATPase activation by ClpP		Enhancement of ClpC unfolding activity by ClpP	
	EM	SEC	EM	SEC	LY-AMC	LY-AMC + ADEP1	FITC-casein + ADEP1	FITC-casein	GFP-SsrA	ClpC/MecA	ΔN-ClpC-R443A	ClpC/MecA-mEOS3.2	ΔN-ClpC-R443A/Kaede-SsrA
WT	++	++	++	++	++	++	++	++	++	++	++	++	++
I8A	–	–	–	–	–	(+)	+	–	–	–	–	–	–
E9A	–	–	(+)	–	–	(+)	+++	–	–	–	–	–	–
T10A	++	++	+	++	(+)	++	+++	++	+(+)	++	+(+)	+(+)	+(+)
T11A	–	–	(+)	(+)	–	+	+++	–	(+)	–	–	–	–
N12A	++	++	++	++	++	++	++	++	++	++	+(+)	++	++
R13A	++	+	++	+	++	++	+	+	+	+	+	+	+
G14A	–	–	–	–	–	++	+++	–	–	–	–	–	–
E15A	(+)	–	++	++	–	++	+++	(+)	+	(+)	+	(+)	+(+)
R16A	(+)	–	–	–	(+)	++	+	(+)	(+)	–	–	–	–

Oligomerization and MecA/ClpC complex formation properties and proteolytic activities of ClpP β-hairpins mutants were qualitatively assessed. The properties and activities of ClpP-WT were defined as "++". ClpP mutants were categorized into three groups: no major defects (green), partial defects (orange) and severe defects (red).

E.coli strains used in this study

Strain	Description	Source
E. coli XL1 blue	*recA1 endA1 gyrA96 thi-1 hsdR1 supE44 relA1 lac [F' proAB lacI^{q} DM15 Tn10 (Tcr)]*	Stratagene
E. coli BL21	*F- ompT lon hsdSB gal dcm l (DE3)*	Novagen
E. coli ΔclpB::kan	MC4100 *ΔclpB::Km* (Kanamycin resistant)	Mogk lab (Katikaridis et al, 2021)
E. coli ΔclpX ΔclpP	MC4100 *ΔclpX ΔclpP*	Bukau lab (Dougan et al, 2002)

Dougan, D. A., Reid, B. G., Horwich, A. L., and Bukau, B. (2002) ClpS, a substrate modulator of the ClpAP machine *Mol Cell* **9**, 673-683.
Katikaridis P, Romling U, Mogk A (2021) Basic mechanism of the autonomous ClpG disaggregase. J Biol Chem: 100460

Uncropped blots for figures and supplementary figures

c

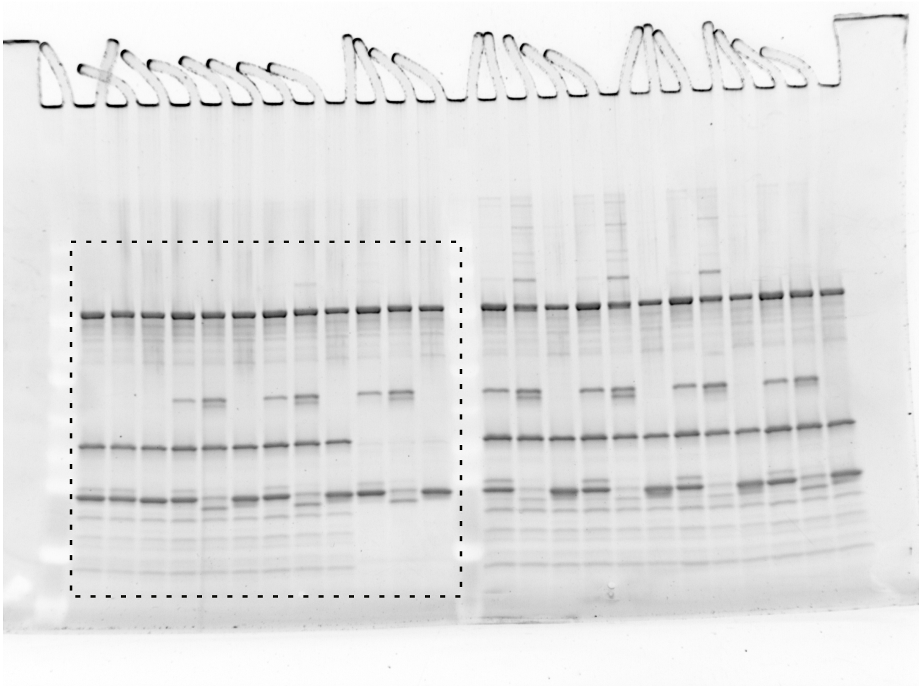

d

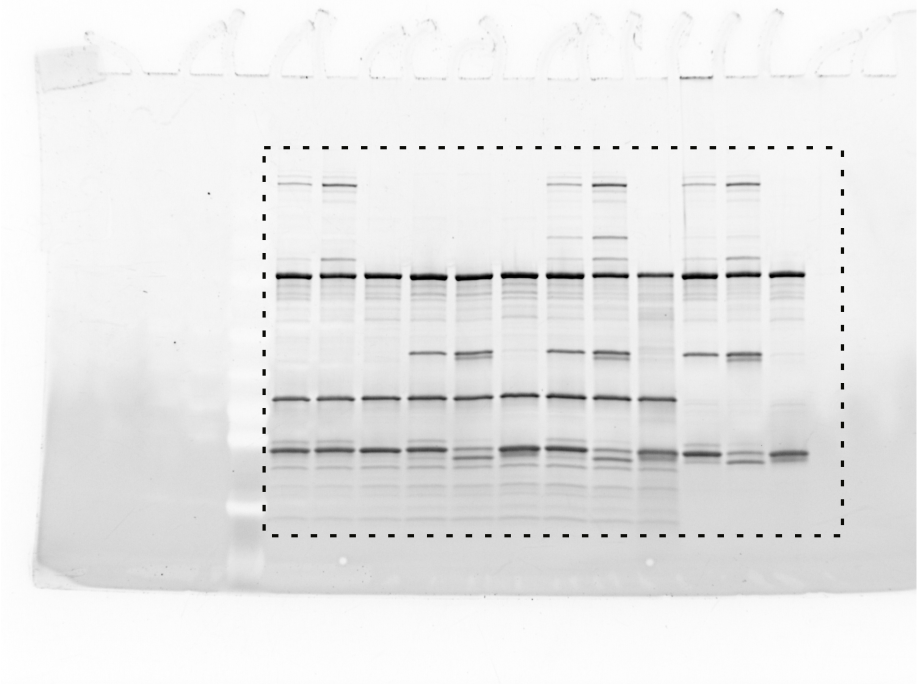

Supplementary Figure 6b

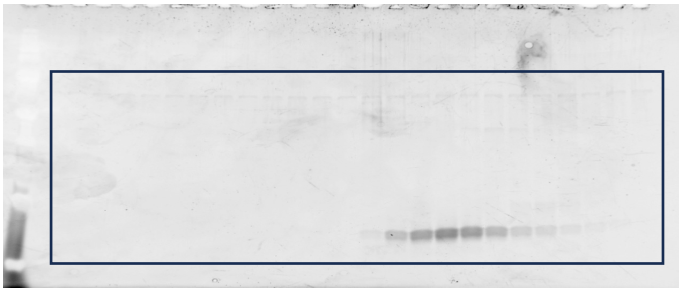

ClpP

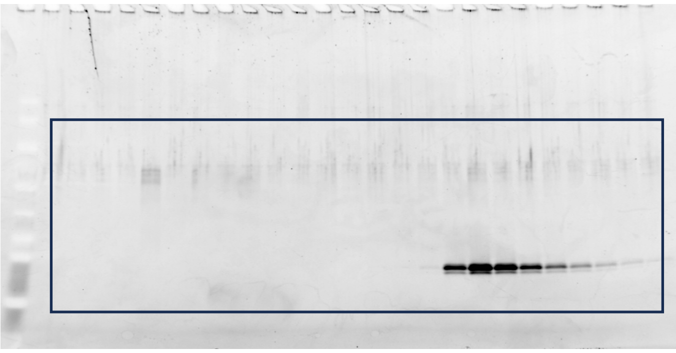

ClpP-I8A

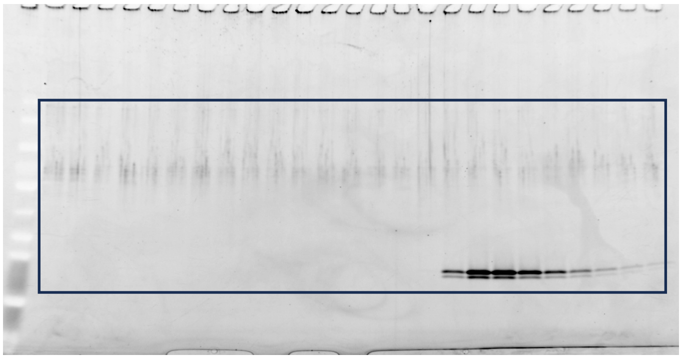

ClpP-E9A

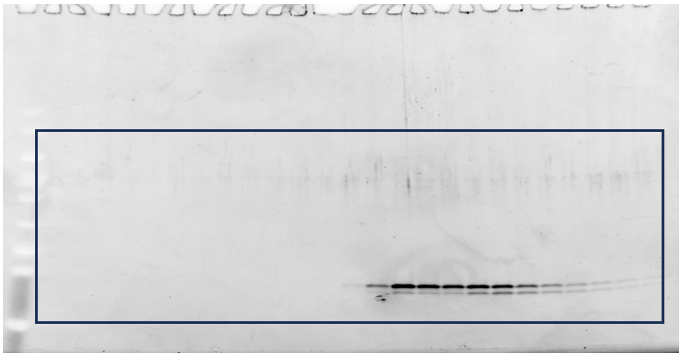

ClpP-T10A

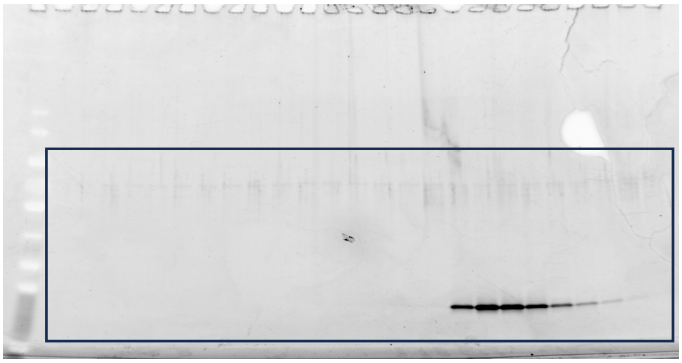

ClpP-T11A

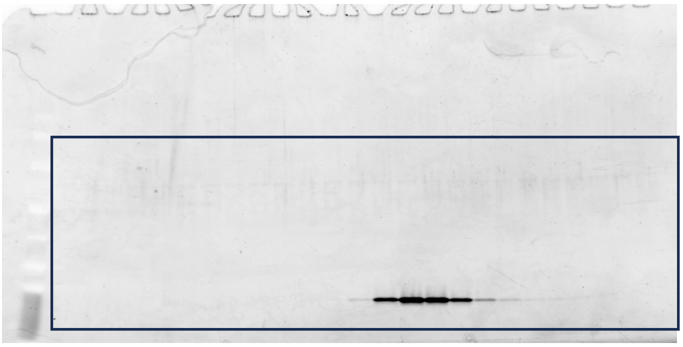

ClpP-N12A

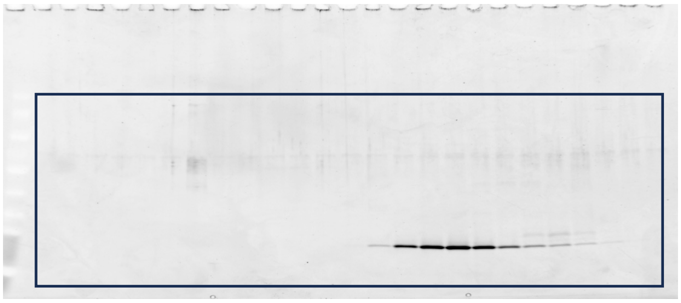

ClpP-R13A

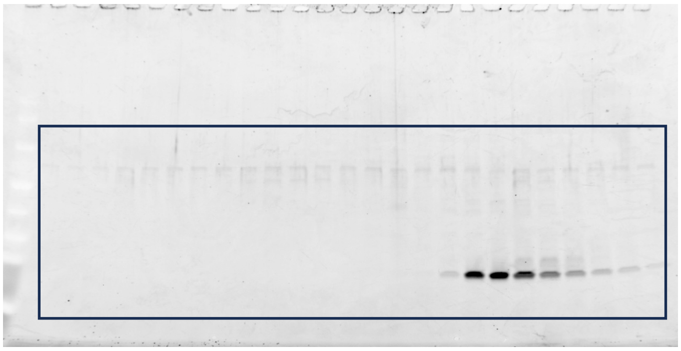

ClpP-G14A

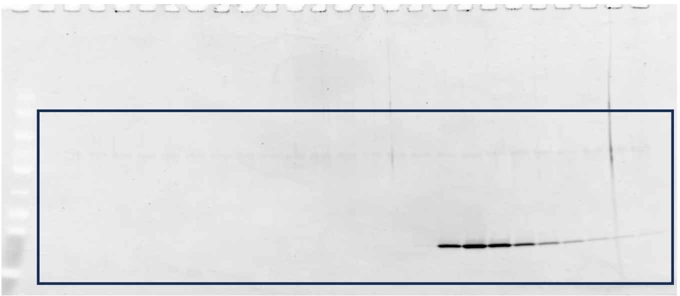

ClpP-E15A

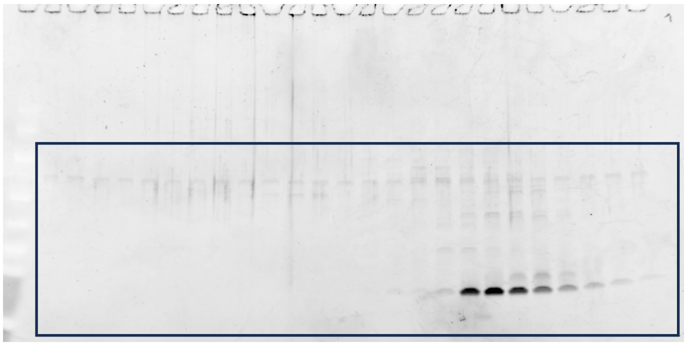

ClpP-R16A

Supplementary Figure 7b

ClpP

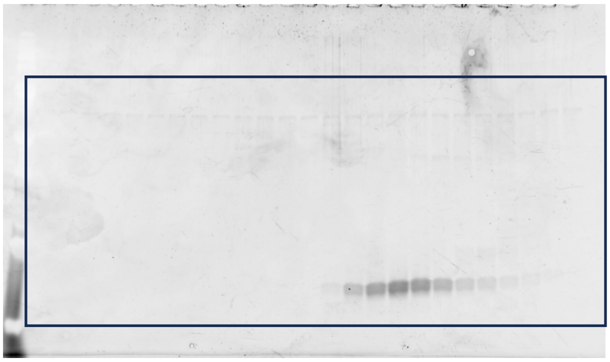

ClpC + MecA + ClpP-E9A

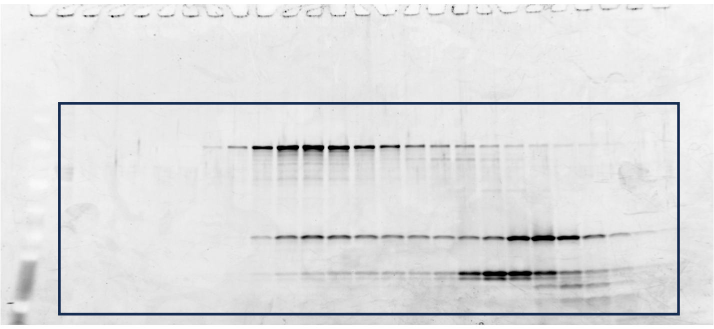

ClpC + ClpP

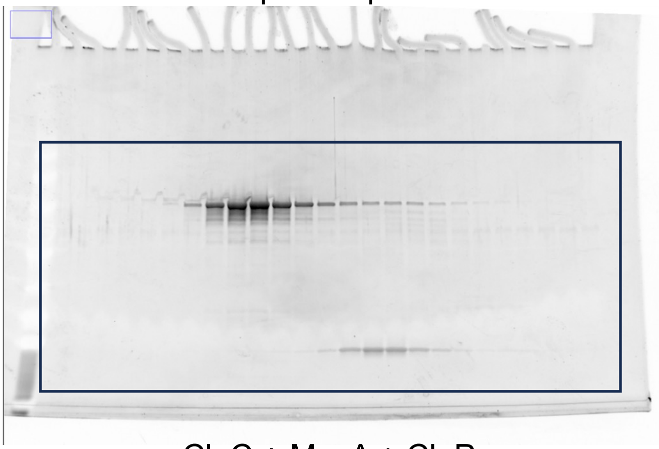

ClpC + MecA + ClpP-T10A

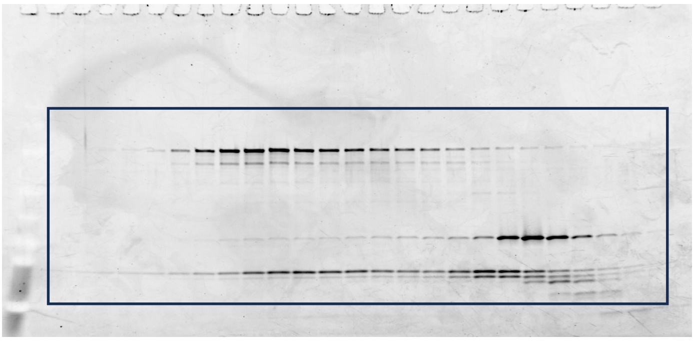

ClpC + MecA + ClpP

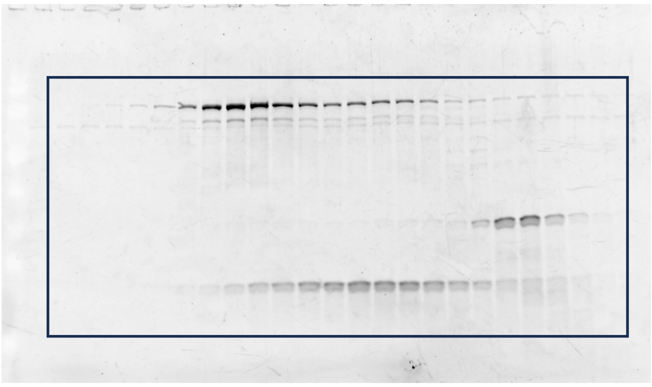

ClpC + MecA + ClpP-T11A

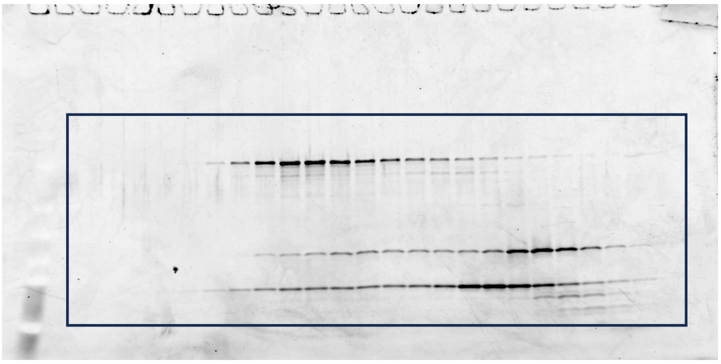

ClpC + MecA + ClpP-I8A

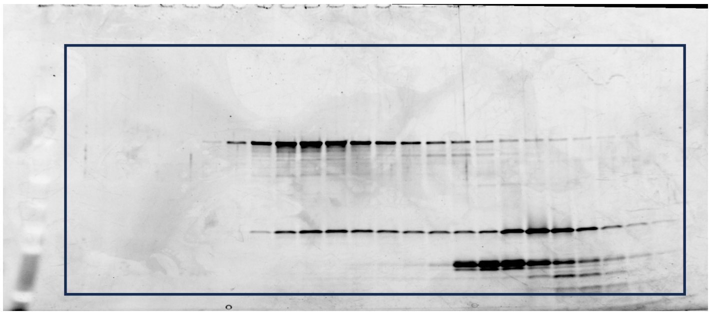

ClpC + MecA + ClpP-N12A

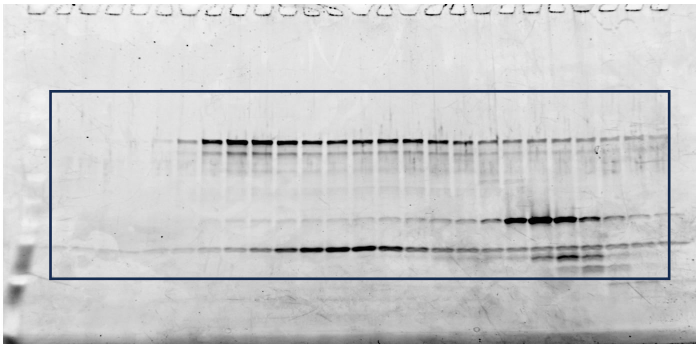

ClpC + MecA + ClpP-R13A

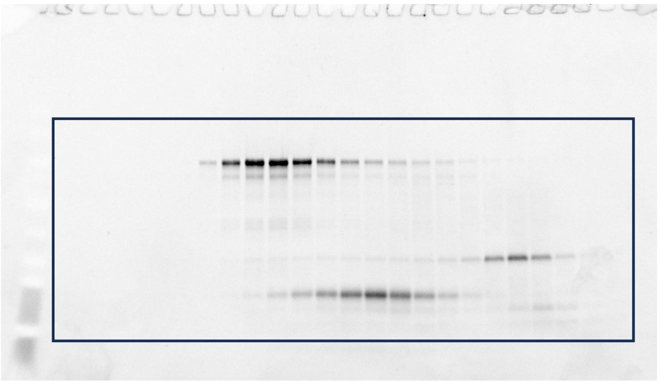

ClpC-G672D/F573D + MecA + ClpP

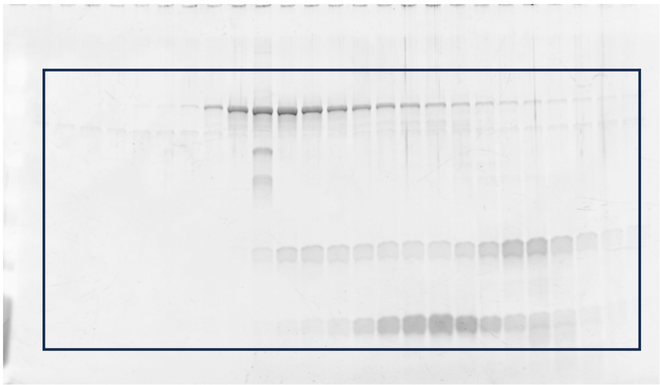

ClpC + MecA + ClpP-G14A

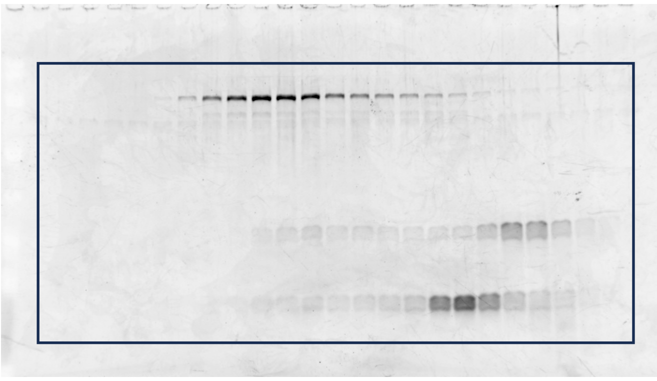

ClpC + MecA + ClpP-E15A

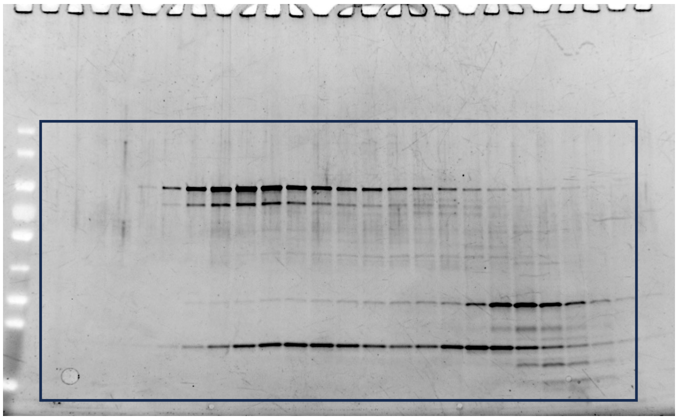

ClpC + MecA + ClpP-R16A

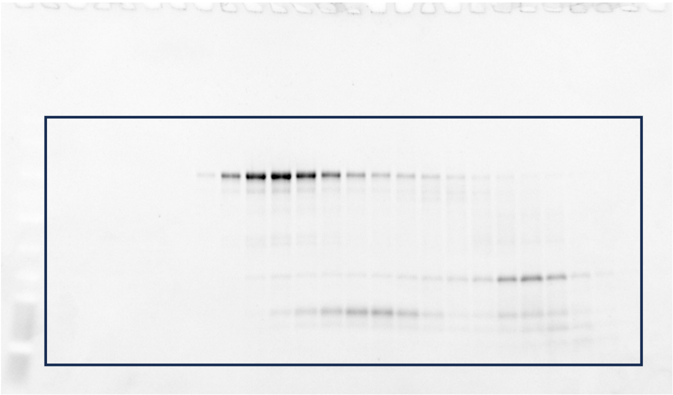

Supplementary Figure 8

b

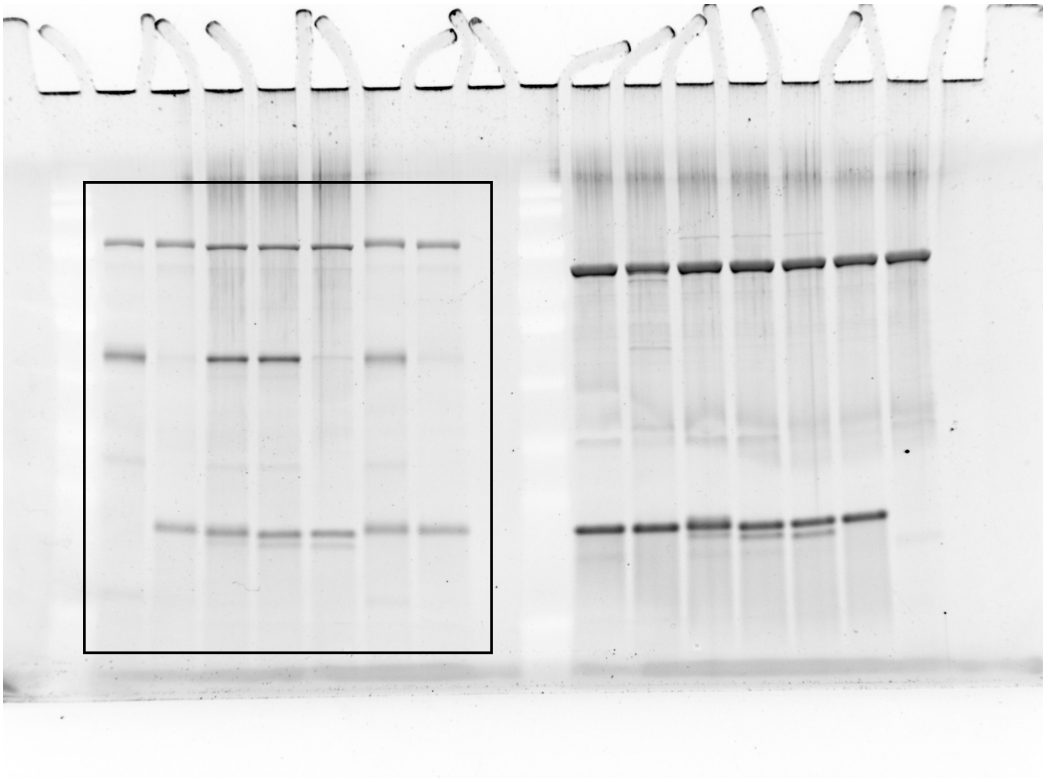

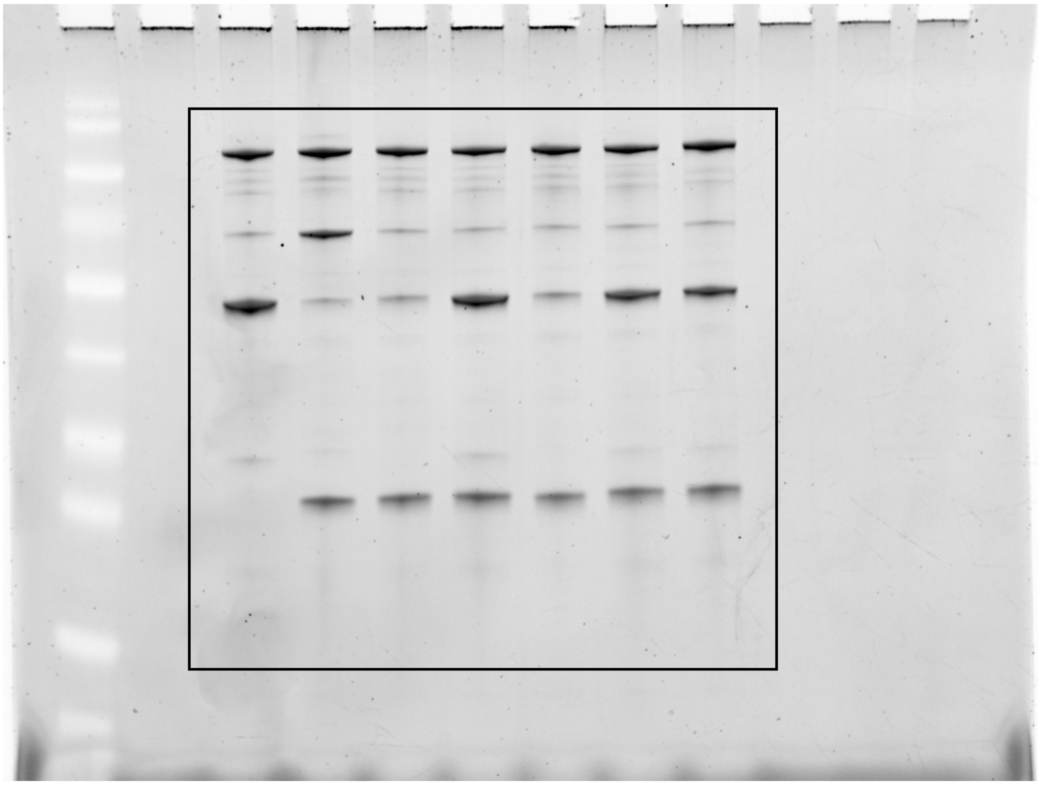

c

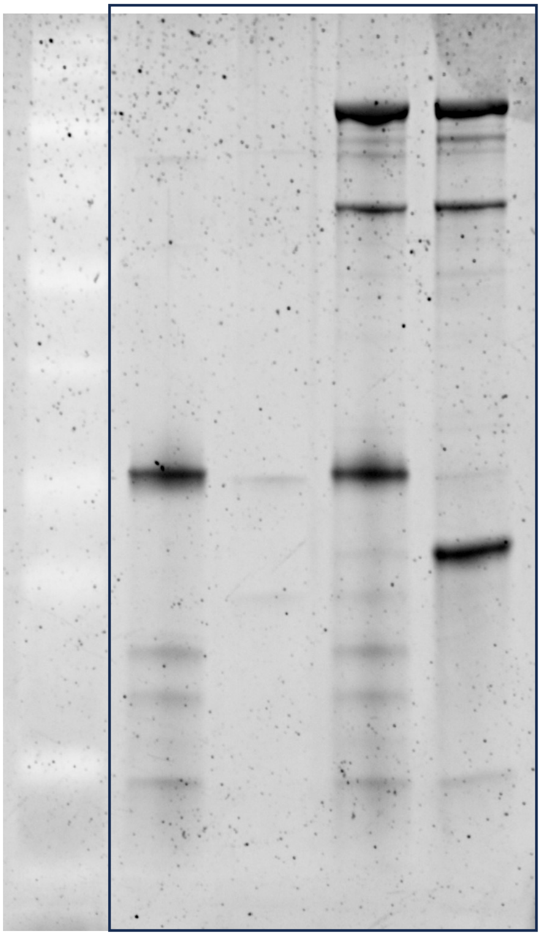

Sypro stained

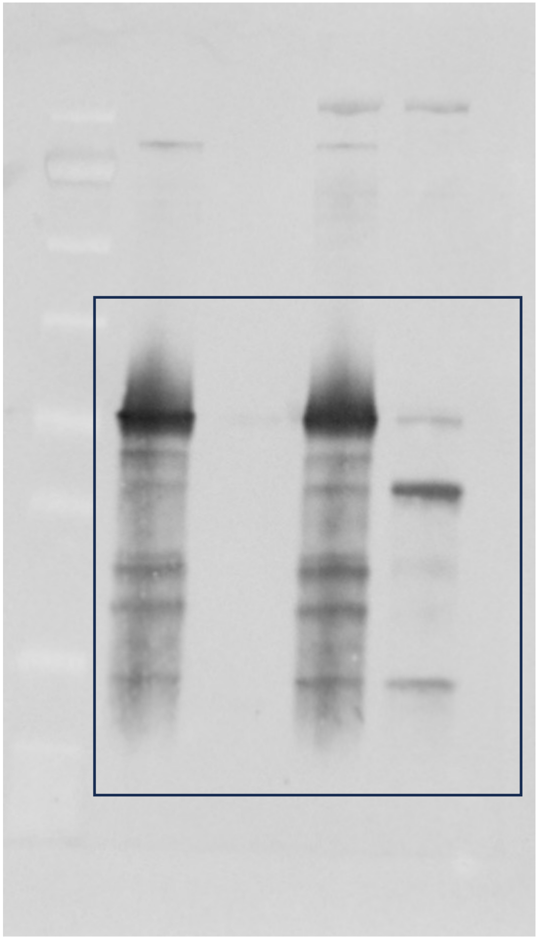

anti-MecA

Supplementary Figure 9

c

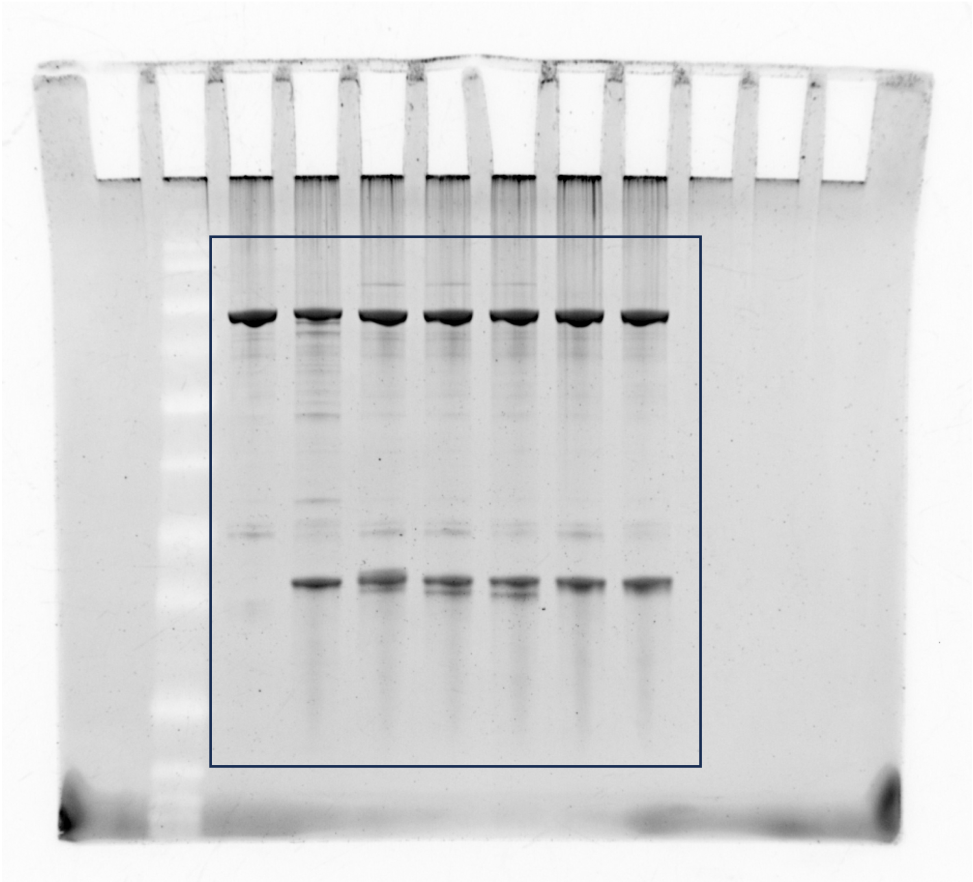

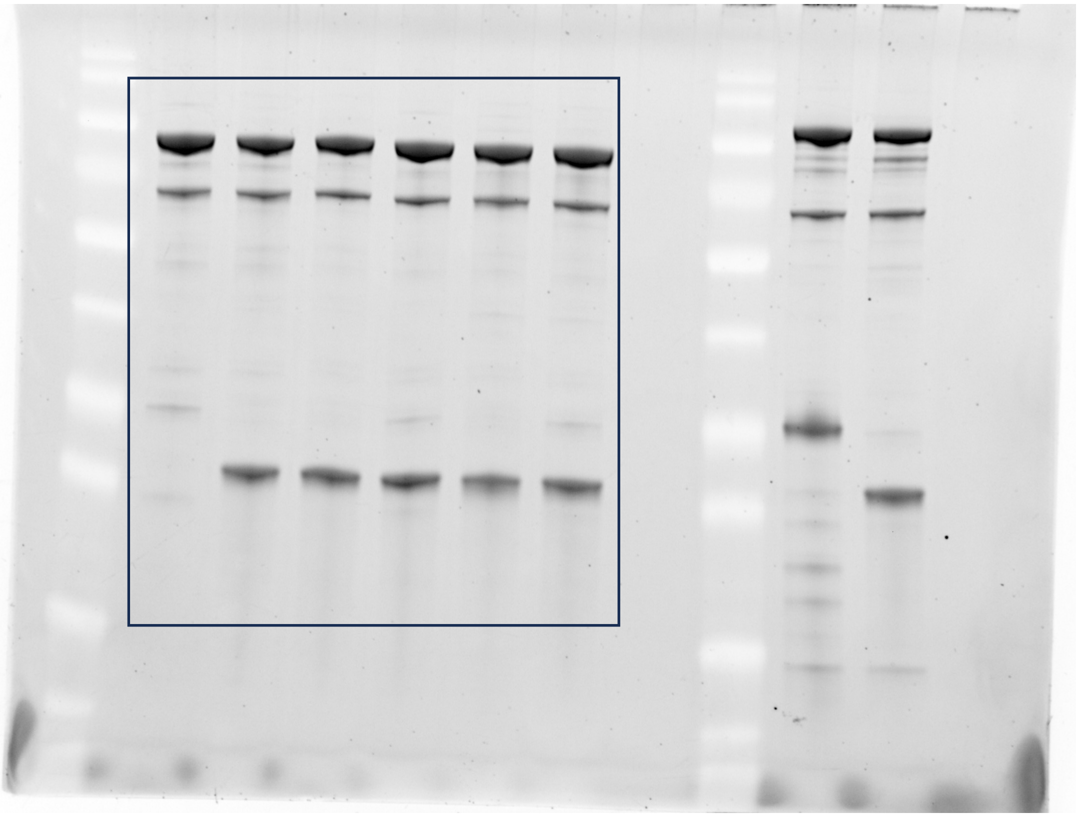

Supplement: Supplementary file 2 — Supplementary information [file 42003_2025_8908_MOESM2_ESM.pdf]
