# Supplementary material for: Structure of the central Staphylococcus aureus AAA+ protease MecA/ClpC/ClpP
Source: Commun Biol. 2025 Oct 14;8:1467. doi: 10.1038/s42003-025-08908-w (PMC12521514; doi:10.1038/s42003-025-08908-w)
Supplement: Supplementary file 3 — Description of Supplementary Files [file 42003_2025_8908_MOESM3_ESM.pdf]

Description of Additional Supplementary Files

File name: Supplementary Data 1

Description: Source data for the Figures

File name: Supplementary Video 1

Description: Motions of the MecA/ClpC/ClpP complex's bodies (MecA crown, ClpC/ClpP and ClpP protease bodies) relative to each other. Animation of the volumes obtained via 3D variability analysis. Suggested to play at 0.5x.
